# Density-dependent role of an invasive marsh grass, *Phragmites australis*, on ecosystem service provision

**DOI:** 10.1371/journal.pone.0173007

**Published:** 2017-02-24

**Authors:** Seth J. Theuerkauf, Brandon J. Puckett, Kathrynlynn W. Theuerkauf, Ethan J. Theuerkauf, David B. Eggleston

**Affiliations:** 1 Department of Marine, Earth and Atmospheric Sciences, Center for Marine Sciences and Technology, North Carolina State University, Morehead City, North Carolina, United States of America; 2 North Carolina Coastal Reserve and National Estuarine Research Reserve, Beaufort, North Carolina, United States of America; 3 Illinois State Geological Survey, University of Illinois at Urbana-Champaign, Champaign, Illinois, United States of America; Shandong University, CHINA

## Abstract

Invasive species can positively, neutrally, or negatively affect the provision of ecosystem services. The direction and magnitude of this effect can be a function of the invaders’ density and the service(s) of interest. We assessed the density-dependent effect of an invasive marsh grass, *Phragmites australis*, on three ecosystem services (plant diversity and community structure, shoreline stabilization, and carbon storage) in two oligohaline marshes within the North Carolina Coastal Reserve and National Estuarine Research Reserve System (NCNERR), USA. Plant species richness was equivalent among low, medium and high *Phragmites* density plots, and overall plant community composition did not vary significantly by *Phragmites* density. Shoreline change was most negative (landward retreat) where *Phragmites* density was highest (-0.40 ± 0.19 m yr^-1^ vs. -0.31 ± 0.10 for low density *Phragmites*) in the high energy marsh of Kitty Hawk Woods Reserve and most positive (soundward advance) where *Phragmites* density was highest (0.19 ± 0.05 m yr^-1^ vs. 0.12 ± 0.07 for low density *Phragmites*) in the lower energy marsh of Currituck Banks Reserve, although there was no significant effect of *Phragmites* density on shoreline change. In Currituck Banks, mean soil carbon content was approximately equivalent in cores extracted from low and high *Phragmites* density plots (23.23 ± 2.0 kg C m^-3^ vs. 22.81 ± 3.8). In Kitty Hawk Woods, mean soil carbon content was greater in low *Phragmites* density plots (36.63 ± 10.22 kg C m^-3^) than those with medium (13.99 ± 1.23 kg C m^-3^) or high density (21.61 ± 4.53 kg C m^-3^), but differences were not significant. These findings suggest an overall neutral density-dependent effect of *Phragmites* on three ecosystem services within two oligohaline marshes in different environmental settings within a protected reserve system. Moreover, the conceptual framework of this study can broadly inform an ecosystem services-based approach to invasive species management.

## Introduction

Invasive species pose significant conservation challenges, and their spread is considered second only to habitat destruction as the greatest cause of species endangerment and global loss of biodiversity [1]. In the United States alone, estimated damages and control costs of invasive species total more than $137 billion per year [2]. Despite extensive efforts, eradication of most invasive species has been largely ineffectual or unresolved and the impact of invasive species on ecosystem service provision remains largely unknown [1]. Positive, neutral, and negative impacts of invasive species on ecosystem service provision have been reported [3–5], with impacts often strongly linked to invasive species density [6]. Given the significant costs associated with eradication measures, coupled with the variable impacts of invasive species on ecosystem service provision, an understanding of the role of invasive species in the provision of essential ecosystem services along a gradient of invasive species density is warranted.

Marsh ecosystems lie at the interface between upland and open water, yielding high levels of primary and secondary production that support terrestrial and aquatic food webs, shoreline stabilization, and carbon storage [7–9]. Recently, there has been considerable interest in the carbon storage services associated with marsh ecosystems, specifically the capacity of marshes to store atmospheric carbon trapped through primary production and vertical marsh accretion, and their ability to protect shorelines through baffling erosive wave energy [9, 10]. A key factor underpinning the ability of marshes to deliver ecosystem services is the diversity and structure of the marsh plant communities, which are often positively correlated with primary production, nutrient cycling, and resiliency to disturbance [11, 12]. Alterations to community composition or diversity through, for instance invasive species outcompeting native plant species, could disrupt the delivery of ecosystem services.

Invasive species are one of the many factors contributing to the loss of marsh ecosystems. An estimated 25% of tidal marshes worldwide have been lost, and the current loss rates in North America are around 1–2% per year [13, 14]. Invasive species, particularly marsh plants, threaten marsh ecosystems by displacing dominant native species. For example, along the Atlantic coast of North America, the invasive marsh grass *Phragmites australis* (hereafter *Phragmites* [15]) has displaced native marsh grasses such as *Spartina alterniflora* and *Spartina cynosuroides* (hereafter *Spartina*). *Phragmites* is a tall (1–4 m) wetland grass with a global distribution that can persist and thrive in a wide range of conditions, ranging from damp soil to standing water. In suitable conditions, such as oligohaline systems (0.5–5 psu), *Phragmites* can spread via rhizomes or seeds at a rate of ≥ 5 m per year and can displace native marsh grasses (e.g., through growth-induced modification of edaphic soil conditions, such as rhizosphere oxygenation and reductions in sulfide concentrations, that promote expansion; [16, 17]). In polyhaline systems (5–18 psu), *Phragmites* is generally less dominant.

Reported effects of *Phragmites* on marsh ecosystem service provision have varied. For example, Chambers *et al*. ([15]) reported no reduction in the capacity of the marsh to support higher trophic levels or reduction of water purification and sediment stabilization with *Phragmites* dominance. Stalter and Baden ([18]) reported a decrease in overall plant species diversity with *Phragmites* presence. Windham ([19]) reported a five-fold increase in annual carbon accumulation rates (three-fold increase aboveground, two-fold increase belowground) in *Phragmites* marshes communities relative to native marsh plant communities. Despite variation in reported impacts of *Phragmites* on marsh ecosystems and associated ecosystem services, many agencies continue attempts to eradicate *Phragmites* to protect native marsh species [20]. *Phragmites* eradication techniques include chemical, mechanical or physical control methods [21]. Despite considerable effort and cost, successful *Phragmites* eradication is often not achieved without multiple follow-up efforts due to survival of *Phragmites* seeds and rhizomes [22].

The quantity and quality of ecosystem services provided by habitat-forming ecosystem engineers (e.g., *Phragmites* and *Spartina* spp.) is often correlated with abundance of the ecosystem engineer [23]. Furthermore, the impacts of invasive species on native biota, community structure, and ecosystem service provision are often strongly density-dependent [6]. While many studies have focused on identifying the impacts of invasive species on native biota and ecosystem attributes, these studies often take the form of comparing treatments with no invasive species present versus high invasive species density ([6] and references therein). Understanding the density-dependent role of invasive species in ecosystem service provision can improve management actions and potentially lower costs by identifying a density threshold above which action is required [24, 6].

This study assessed the density-dependent role of *Phragmites* on the provision of three estuarine marsh ecosystem services—shoreline stabilization, carbon storage, and plant diversity—by quantifying (i) shoreline change rates, (ii) below-ground carbon inventory, and (iii) plant species diversity and community structure along a *Phragmites* density gradient. The ecosystem services were quantified along a *Phragmites* density gradient at two reserves within the North Carolina Coastal Reserve and National Estuarine Research Reserve (NCNERR) that vary in salinity and shoreline wave exposure. The present study provides a framework for utilizing ecosystem service provision as the basis for developing a density-dependent threshold for *Phragmites* management, and this conceptual framework has broad implications for global management of invasive species using density-and ecosystem services-based approaches.

## Methods

### Study area

**Fig 1:**
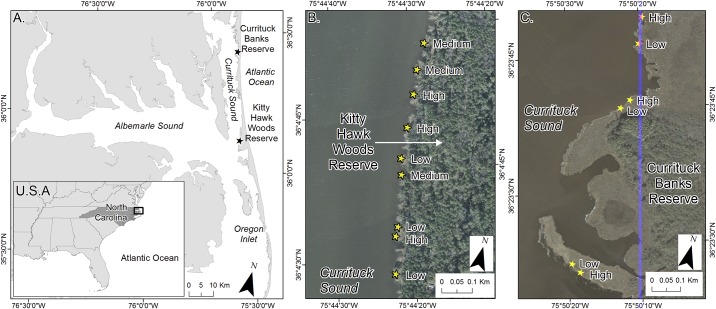
Sampling locations. A) Location of Currituck Banks and Kitty Hawk Woods Reserves (stars), components of the North Carolina Coastal Reserve and National Estuarine Research Reserve system, in Currituck Sound, North Carolina, USA. B) Location of sampled Low, Medium, and High *Phragmites* Density sites (stars) within Kitty Hawk Woods Reserve. C) Location of sampled Low and High *Phragmites* Density sites (stars) within Currituck Banks Reserve—note that no Medium *Phragmites* density sites were present within Currituck Banks Reserve. All satellite imagery was derived from United States Geological Survey, High Resolution Orthoimagery Dataset.

We assessed the density-dependent role of *Phragmites* on ecosystem service provision at Currituck Banks and Kitty Hawk Woods Reserves, which are two Reserves within the NCNERR located in Currituck Sound, North Carolina (Fig 1). These Reserves were selected because: (1) they represent the range of salinities observed within Currituck Sound, with Kitty Hawk Woods Reserve exhibiting a higher mean salinity (3.6) than Currituck Banks Reserve (1.3 [25]), (2) shoreline fetch distances and corresponding wave exposure varied between reserves, with greater distances at Kitty Hawk Woods Reserve (5–30+ km fetch) than Currituck Banks Reserve (5–10 km fetch), and (3) a gradient in density of *Phragmites* and native marsh vegetation (i.e., *Spartina*) was available for both Reserves. The long-term history of *Phragmites* presence in the marshes of these Reserves is largely unknown, although mapping of *Phragmites* extent and spread began in 1980 (Scott Crocker, NCNERR site manager, pers comm). Permission to conduct field work within the two Reserves within the NCNERR was granted by the North Carolina Coastal Reserve and National Estuarine Research Reserve Research Permit #6–2015.

Currituck Sound is a brackish-water estuary that receives freshwater input from adjacent tributaries and Albemarle Sound, and saltwater input from the Atlantic Ocean via Oregon Inlet. Oceanic tidal influence is negligible, although saltwater intrusion via Oregon Inlet yields a more variable salinity nearest Kitty Hawk Woods Reserve (ranging from 0.3–20.3 psu), as compared to the more stable salinities nearest Currituck Banks Reserve (ranging from 0.5–3.6 psu [25]). Urban development and shoreline hardening is pervasive along the eastern shoreline of Currituck Sound, although there is no shoreline hardening present within the Reserve sites examined in this study [26].

### Quantifying marsh ecosystem services

We quantified the density-dependent role of *Phragmites* on the following ecosystem service response variables: (i) shoreline change rates, (ii) below-ground carbon inventory, and (iii) plant species diversity and community structure. To do so, we sampled 9 randomly selected marsh sites within Kitty Hawk Woods Reserve (3 sites within Low, Medium, and High *Phragmites* Density treatment categories, described below; Fig 1B), and 6 randomly selected sites within Currituck Banks Reserve (3 sites with Low and High *Phragmites* Density treatment categories, described below; Fig 1C). Prior to site selection during spring 2015, we conducted a ground-truth survey of existing distribution maps for each Reserve to identify: (1) Low *Phragmites* Density sites where the dominant native marsh grass, *Spartina*, was most abundant (> 40% cover m^-2^) and *Phragmites* was least abundant (< 1%), (2) Medium *Phragmites* Density sites with mixed vegetation where native marsh grass (~ 20% cover) and *Phragmites* (~ 40% cover) are present together, and 3) High *Phragmites* Density sites where *Phragmites* was most abundant (> 40%) and *Spartina* was least abundant (< 5%). Sampled sites were randomly selected from the pool of potential sites within each reserve that were identified from the ground-truth survey. Based on the initial ground-truth survey, we were unable to locate any Medium *Phragmites* Density sites at Currituck Banks Reserve, thus the study focused on sites within Low and High *Phragmites* Density categories for Currituck Banks Reserve.

#### Shoreline change rates

We installed shoreline position benchmarks to quantify marsh shoreline change rates (sensu [27]). Shoreline position benchmarks, as described in greater detail below, provide stable, fixed reference points from which changes in shoreline position can be evaluated over time. In May 2015, we installed between 3–5 iron-rebar stake pairs by driving two stakes (9.5 mm diameter, 1.22 m length) ~ 1 m into the ground at each site for each *Phragmites* Density treatment in both Reserves. We determined the number of shoreline position benchmarks assigned to a site based on the shoreline length of a given site, with the longest shoreline lengths receiving 5 pairs of equally spaced (~ 15 m apart) rebar stakes, and the shortest receiving 3 pairs of equally spaced rebar stakes. The first rebar stake was installed approximately 2 m soundward of the shoreline. The second rebar stake was installed perpendicular to the shoreline from the first rebar approximately 1 m landward of the first *Phragmites* or *Spartina* plants. Care was taken to ensure minimal impact and damage to marsh plants in the area of the shoreline position benchmarks. The survey tape was pulled taut between the two rebar stakes, and the distance from the in-marsh stake to the marsh edge (i.e., the interface between the vegetated marsh surface and shallow open water) was measured to establish a shoreline distance baseline. One year later, in May 2016, we returned to the sites to repeat the measurements from the in-marsh stake to the marsh edge to determine shoreline changes (i.e., landward (negative) or soundward (positive)) in the marsh edge measured as m yr^-1^. We compared these rates of shoreline change with historical rates of shoreline change estimated from aerial imagery collected for these sites during 1996 and 2012 at Kitty Hawk Woods and 2003 and 2012 at Currituck Banks (NCNERR unpubl. data, [26]). Historical shoreline changes were measured by digitizing the shoreline within ArcGIS software and using the measurement tool to quantify shoreline change at each of the shoreline position benchmark sites monitored in our study [28]. We divided the distance change between shoreline position at *t*_*1*_ versus shoreline position at *t*_*2*_ by the number of years between the two shorelines to determine an annual rate in m yr^-1^.

We compared mean shoreline change rates (m yr^-1^) between Reserves, *Phragmites* Density treatments, and their interaction using a two-way ANOVA, and subsequently conducted one-way ANOVA for comparisons within the individual factors. Data were tested for homogeneity of variance using Levene’s Test. Non-transformed and transformed data failed to meet the assumption of homoscedasticity, however, further examination of residual plots of non-transformed data indicated that they were approximately normal and therefore were used in data analysis due to the robustness and insensitivity of ANOVA to skewness [29]. For comparison of shoreline change rates among reserves, only data for Low and High *Phragmites* Density treatments was included as the Medium *Phragmites* Density treatment was not present at both Reserves. Where appropriate, comparisons among treatments within a factor were conducted with a Tukey-Kramer multiple comparisons test.

#### Below-ground carbon inventory

To quantify the below-ground carbon inventory, we collected sediment push-cores for organic carbon content analysis and carbon inventory calculations [30, 14]. Push cores were collected from one randomly selected site (out of three possible) within each of the *Phragmites* Density treatments in each Reserve (Low, Medium and High Density for Kitty Hawk Woods and Low and High Density for Currituck Banks). We used a randomly selected shoreline position benchmark (e.g. rebar stake; see shoreline change rates above) at each selected site as a reference to then extend a transect tape from the marsh edge to the upland marsh and forest transition zone (mean distance = 19.53 m, SD = 5.11 m). Along each transect, we collected three ~75 mm diameter, sediment push-cores for a total of 9 sediment cores from Kitty Hawk Woods and 6 cores from Currituck Banks (Fig 1). Cores were obtained from the marsh edge, the upland marsh and forest transition zone (~20 m from the marsh edge), and the midpoint between these two locations (~10 m from the marsh edge). Cores were driven to a depth of refusal, which was generally ~0.5 m. Sampling was conducted in late October 2015, which corresponds with the post-growing season for marshes in our study area ([31], sensu [32]). It is important to note that below-ground carbon inventory estimates are relatively insensitive to the timing of sampling within a given year as the accumulated sediments retained within core samples integrate over decadal time-scales [14].

In the laboratory, each core was split in half, photographed, and described to determine the marsh depth boundary (defined as the contact between the organic-rich marsh unit and the underlying sandy unit). One half of the core was preserved via freezing and the other half was sampled at 5 cm intervals from the top of the core to the marsh depth boundary. The sampled material from each 5 cm interval was dried at 60°C for 48 hr. Total weight of the dried sediment was determined to the nearest tenth of a gram. The dried sediment was then placed into a food processor and finely ground until all material within the sample was homogenized. Approximately 5 ml of the sample was decanted into a labeled vial for subsequent organic carbon content analysis. A total of 100 randomly selected samples (out of 201 total samples) were analyzed for organic carbon content on a Costech ECS4010 Elemental Analyzer (CHN) at the University of North Carolina at Chapel Hill Institute of Marine Sciences. All 201 samples were processed via loss-on-ignition (LOI) analysis (i.e., combustion of ~ 5 ml samples at 450°C for 4 hr in a muffle furnace) to determine percent organic material [33].

Linear regression models were used to predict the relationship between percent organic material (determined via LOI) and percent organic carbon content (determined via CHN) so that we could convert percent organic material of the 101 not analyzed using CHN into percent organic carbon content (sensu [31]). We used likelihood ratio tests [34] to evaluate differences in these relationships among Reserves, and *Phragmites* Density. From this analysis, regression functions did not differ by Reserve or *Phragmites* Density (χ^2^ ≤ 3.9, df = 2, p ≥ 0.14), yet differed by the Reserve x *Phragmites* Density interaction (χ^2^ = 10.9, df = 2, p = 0.004). Thus, we generated Reserve- and *Phragmites* Density-specific LOI versus organic carbon content relationships (S1 Fig). Given the lower cost of processing samples via LOI relative to CHN, these models provide a cost-effective means of quantifying percent organic carbon in marsh sediments and are the first *Phragmites*-specific LOI to organic carbon content models we are aware of.

For each 5 cm core sample, we used the total weight of the dried sediment (g) and the associated percent organic carbon to determine the organic carbon content (g). For all core sections with > 1% organic carbon content, we summed the organic carbon content across sections and scaled the total by the inverse of the area of the sediment push-core to determine total below-ground carbon inventory in g carbon m^-2^ [14]. As the thickness of the marsh unit varied among cores from different *Phragmites* Density treatments, we also normalized the carbon inventories by dividing by the marsh unit depth, yielding standardized units of g carbon m^-3^, to draw direct comparisons of below-ground carbon inventories between Reserves and *Phragmites* Density treatments.

We compared mean, normalized below-ground carbon inventories (g carbon m^-3^) between Reserves and *Phragmites* Density treatments using a two-way ANOVA, and subsequently conducted one-way ANOVA for comparisons within the individual factors. Where appropriate, comparisons among treatments within a factor were conducted with a Tukey-Kramer multiple comparisons test.

#### Plant diversity, community structure, and above-ground biomass

At each site in all *Phragmites* Density treatments in both Reserves, a transect tape was placed perpendicular to the shoreline edge and extended from the marsh edge to the upland marsh and forest boundary. Vegetation survey transects were established adjacent to the shoreline position benchmarks (see ‘Shoreline change rates’ above). The number of transects at a given site was based on the number of shoreline benchmarks at the site (i.e., 3–5 transects were run per site). Sample plots were evaluated every 3–5 m along the transect; a minimum of 3, and as many as 5, sample plots were evaluated per transect. At each sample plot, a 1 m^2^ quadrat was laid down and a 0.25 m^2^ quadrat was placed in the top-left interior of the 1 m^2^ quadrat. Sampling was conducted in August 2015, which corresponds with the peak of annual marsh plant productivity for our study area ([35], sensu [32]).

#### Plant diversity and community structure

Within the 1 m^2^ quadrat, we assessed percent cover of plant species using the North Carolina Vegetation Survey percent cover categorical method [36]. We pooled sample plots to evaluate differences in species richness, diversity, and evenness between Reserves and *Phragmites* Density treatments (e.g., Low, Medium and High). We compared species richness (*d*), Pielou’s evenness (*J’*), Shannon diversity (*H’*), and Simpson’s diversity (1-*D*) between Reserves and *Phragmites* Density treatments with four separate two-way ANOVAs. Furthermore, we utilized a two-way crossed analysis of similarities (ANOSIM) and non-metric multidimensional scaling (NMDS) to evaluate the effects of Reserve and *Phragmites* Density on overall plant community structure within PRIMER v7 software [37]. Details regarding ANOSIM and NMDS analyses are included in S1 Text. Note that because inherent differences exist in the dominant marsh plant species across the *Phragmites* densities (i.e., Low *Phragmites* Density treatment has high abundances of *Spartina*, whereas High *Phragmites* Density treatment has high abundances of *Phragmites*), we removed *Phragmites* and *Spartina* percent cover estimates from ANOSIM and NMDS analyses. This was done to evaluate the role of Reserve and *Phragmites* Density on overall plant community structure in the absence of the dominant marsh plant species (i.e., to determine if unique plant communities are associated with varying densities of *Phragmites*).

#### Above-ground biomass

We evaluated differences in above-ground biomass between *Phragmites* Density treatments and Reserves, and compared these differences with below-ground carbon inventory estimates. Within the 0.25 m^2^ quadrat, we measured total stem density and shoot height (m) of 10 representative shoots of *Phragmites* or *Spartina* (for Medium *Phragmites* Density plots, we measured 5 of both *Phragmites* and *Spartina*). We haphazardly collected and measured 10 representative shoots along one transect within each *Phragmites* Density treatment in each Reserve. Shoots were stored in 8-liter plastic zipper bags for subsequent drying (60°C for 48 hr) to generate a height (m) versus dry mass (g) relationship. We established the height-weight relationships for both *Phragmites* and *Spartina* in each *Phragmites* Density treatment at both reserves. We used a global curve-fitting program [38] to fit various functions (e.g., linear and exponential) to the relationships and calculated AICc (second-order bias correction estimator for Akaike’s information criterion) to verify the best fitting model of the possible functions. We used likelihood ratio tests (Kimura 1980) to evaluate differences in these relationships among Plant Species (*Phragmites* vs. *Spartina*), Reserves, and *Phragmites* Density. From this analysis, functions varied by Plant Species and *Phragmites* Density (χ^2^ ≥ 47.2, df = 2, p ≤ 5.5e-11), but not by Reserve (χ^2^ = 0.6, df = 2, p = 0.7), thus we pooled data to generate Plant Species- and *Phragmites* Density-specific shoot height versus dry mass relationships (S2 Fig). Using information on per-unit area mean stem density and mean height, we scaled the corresponding height versus dry mass relationship to determine per-unit area above-ground biomass estimates. We compared mean above-ground biomass between Reserves and *Phragmites* Density treatments using a two-way ANOVA, and subsequently conducted one-way ANOVA for comparisons within the individual factors. Where appropriate, comparisons among treatments within a factor were conducted with a Tukey-Kramer multiple comparisons test.

## Results

### Shoreline change rates

**Fig 2:**
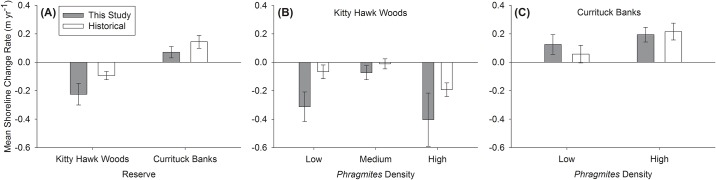
Mean (±SE) shoreline change rate (m yr^-1^) as determined in this study (gray shading) and from historical shoreline imagery (white shading). A) at Kitty Hawk Woods and Currituck Banks Reserves, B) between *Phragmites* Density treatments within Kitty Hawk Woods Reserve, and C) between *Phragmites* Density treatments within Currituck Banks Reserve. Note that the Medium *Phragmites* Density treatment was present only in Kitty Hawk Woods Reserve. Negative values indicate landward retreat and positive values indicate soundward advance. See text for results of statistical analyses.

Mean shoreline change rates varied significantly by Reserve (F_1,41_ = 10.5, p = 0.002), but not by *Phragmites* Density (F_1,41_ = 0.7, p = 0.4), or the interaction of Reserve and *Phragmites* Density (F_1,41_ = 0.1, p = 0.7). Across the study period (1 year), marsh shorelines within Kitty Hawk Woods Reserve retreated landward an average of -0.22 ± 0.08 SE m (relative to -0.09 ± 0.03 m yr^-1^ from historical shoreline imagery), while marsh shorelines within Currituck Banks Reserve advanced soundward an average of 0.07 ± 0.04 m (historical rate: 0.14 ± 0.05 m yr^-1^; Fig 2a). Marsh shoreline change rates in Kitty Hawk Woods Reserve were most negative within the High *Phragmites* Density treatment (-0.40 ± 0.19 m yr^-1^, historical rate: -0.19 ± 0.05 m yr^-1^), moderate within the Low *Phragmites* Density treatment (-0.31 ± 0.10 m yr^-1^, historical rate: -0.07 ± 0.05 m yr^-1^), and least negative within the Medium *Phragmites* Density treatment (-0.07 ± 0.05 m yr^-1^, historical rate: -0.01 ± 0.04 m yr^-1^), although differences were not significant across treatments (all p > 0.05; Fig 2b). Within Currituck Banks Reserve, marsh shoreline change rates were most positive within the High *Phragmites* Density treatment (0.19 ± 0.05 m yr^-1^, historical rate: 0.22 ± 0.06 m yr^-1^) relative to the Low *Phragmites* Density treatment (0.12 ± 0.07 m yr^-1^, historical rate: 0.06 ± 0.06 m yr^-1^), although differences were not significant across treatments (Fig 2c).

### Below-ground carbon inventory

**Fig 3:**
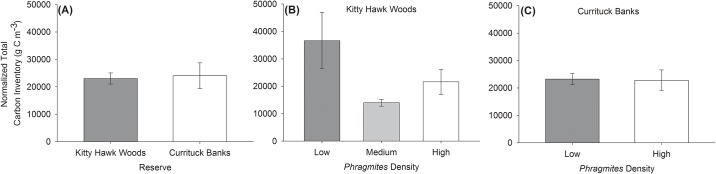
Mean (±SE) normalized total below-ground carbon inventory (g C m^-3^). Below-ground carbon inventory A) at Kitty Hawk Woods and Currituck Banks Reserves, B) between *Phragmites* Density treatments within Kitty Hawk Woods Reserve, and C) between *Phragmites* Density treatments within Currituck Banks Reserve. Note that the Medium *Phragmites* Density treatment was present only in Kitty Hawk Woods Reserve. See text for results of statistical analyses.

Mean normalized below-ground carbon inventory did not vary significantly by Reserve (F_1,14_ = 1.6, p = 0.2), *Phragmites* Density (F_1,14_ = 1.4, p = 0.3), nor the interaction of Reserve and *Phragmites* Density (F_1,14_ = 2.3, p = 0.2). Mean below-ground carbon inventory within Kitty Hawk Woods Reserve was approximately equivalent to that of Currituck Banks Reserve (23,020.03 ± 2,029.25 g C m^-3^ versus 24,074.22 ± 4,647.92 g C m^-3^; p = 0.25; Fig 3a). Although not significant (all p > 0.05), within Kitty Hawk Woods Reserve, mean below-ground carbon inventory was greatest within the Low *Phragmites* Density treatment (i.e., native *Spartina* marsh, 36,627.65 ± 10,220.44 g C m^-3^) relative to the Medium *Phragmites* Density treatment (13,986.2 ± 1,230.71 g C m^-3^) and High *Phragmites* Density treatment (21,608.82 ± 4,533.15 g C m^-3^; Fig 3b). Within Currituck Banks Reserve, mean below-ground carbon inventory was approximately equivalent between Low and High *Phragmites* Density treatments (23,229.48 ± 2,005.59 g C m^-3^ versus 22,810.6 ± 3,752.15 g C m^-3^; p = 0.924; Fig 3c).

### Plant diversity, community structure, and above-ground biomass

**Fig 4:**
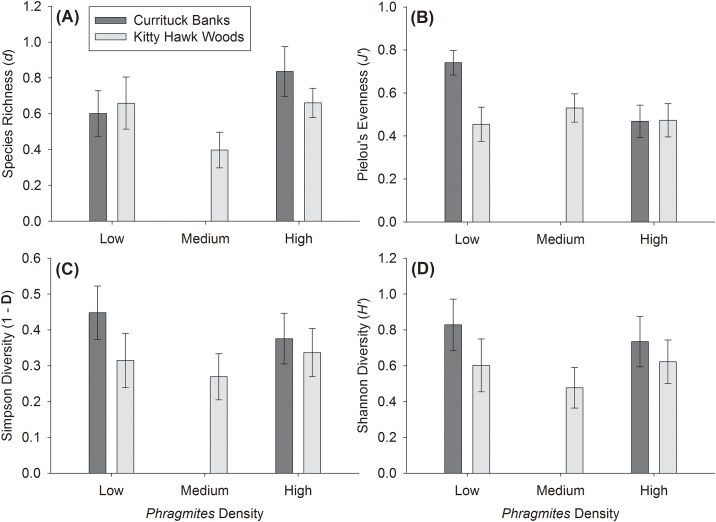
Mean (±SE) observed plant diversity parameters. A) Species Richness (*d*), B) Pielou’s Evenness (*J’*), C) Simpson’s Diversity (1-**D**), and D) Shannon Diversity (*H’*) for Low, Medium, and High *Phragmites* Density treatments in Currituck Banks Reserve (dark gray shading) and Kitty Hawk Woods Reserve (light gray shading). Note that the Medium *Phragmites* Density treatment was present only in Kitty Hawk Woods Reserve. See text for results of statistical analyses.

**Fig 5:**
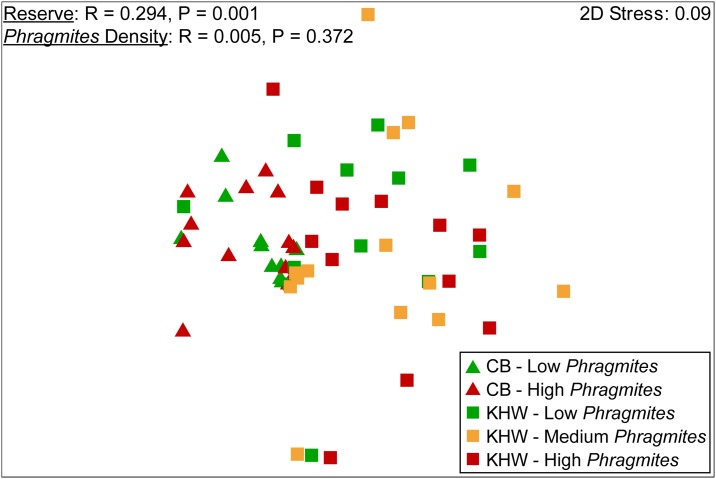
Results of Non-Metric Multidimensional Scaling (NMDS) and two-way crossed Analysis of Similarities (ANOSIM) used to evaluate effects of Reserve and *Phragmites* density on overall emergent vegetation community structure. 2-dimensional stress values denote the degree of mismatch between the predicted values from the regression of the similarity matrix and the distances between samples as displayed by the two-dimensional nMDS plot.

**Fig 6:**
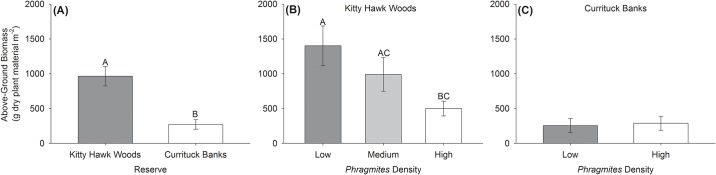
Mean (±SE) above-ground biomass (g dry plant material m^-2^). Above-ground biomass A) at Kitty Hawk Woods and Currituck Banks Reserves, B) between *Phragmites* Density treatments within Kitty Hawk Woods Reserve, and C) between *Phragmites* Density treatments within Currituck Banks Reserve. Note that the Medium *Phragmites* Density treatment was present only in Kitty Hawk Woods Reserve. Letters indicate significant differences between levels of a factor. See text for results of statistical analyses.

Species richness, evenness, Simpson’s diversity index, and Shannon' diversity index did not vary significantly by Reserve (all F_1,46_ < 3.0, p > 0.09), *Phragmites* Density (all F_1,46_ < 2.6, p > 0.1), or the interaction of Reserve and *Phragmites* Density (all F_1,46_ < 3.8, p > 0.06; Fig 4). Overall plant community composition varied significantly by Reserve (R = 0.294, p = 0.001), but not by *Phragmites* Density (R = 0.005, p = 0.37; Fig 5). Mean above-ground biomass varied significantly by Reserve (F_1,46_ = 15.5, p = 0.0003), *Phragmites* Density (F_1,46_ = 6.9, p = 0.01), and the interaction of Reserve and *Phragmites* Density (F_1,46_ = 7.3, p = 0.009). Mean above-ground biomass within Kitty Hawk Woods Reserve was significantly higher (~3.5x higher, 963.78 ± 139.72 SE g dry plant material m^-2^ versus 271.46 ± 69.59) than in Currituck Banks Reserve (F_1,48_ = 12.3, p = 0.001; Fig 6a). Within Kitty Hawk Woods Reserve, mean above-ground biomass decreased with increasing *Phragmites* density. Mean above-ground biomass was significantly higher within the Low *Phragmites* Density treatment (1400.89 ± 285.11 g) than the High *Phragmites* Density treatment (499.56 ± 105.80 g, p < 0.02), but above-ground biomass did not vary significantly between the Medium *Phragmites* Density treatment (988.91 ± 242.83 g) and either the Low or High *Phragmites* Density treatments (all p > 0.28; Fig 6b). Within Currituck Banks Reserve, mean above-ground biomass did not significantly vary between the Low (254.8 ± 101.20 g) and the High *Phragmites* Density treatments (288.12 ± 99.79 g, p = 0.817, Fig 6c).

## Discussion

We identified an overall neutral effect of *Phragmites* density on three important marsh ecosystem services—shoreline stabilization, carbon storage, and plant diversity—in two oligohaline marshes within the North Carolina Coastal Reserve and National Estuarine Research Reserve System (NCNERR), USA. Numerous studies have examined the role of *Phragmites* in the provison of individual marsh ecosystem services [18, 39], but none that we are aware of have simultaneously quantified multiple marsh ecosystem services across a *Phragmites* density gradient at multiple locations within an estuary. Our study broadly highlights the need to: (1) critically evaluate what particular ecosystem services should be preserved when making decisions regarding invasive species management, and (2) conduct on-site monitoring of ecosystem services of management interest along a gradient of invasive species density to determine if density-dependent considerations should be integrated into management plans ([40] and references therein).

The ability of marsh vegetation to protect shorelines from erosion is a critical ecosystem service for natural and human communities and underpins the increased interest in using ‘living shorelines’ for shoreline stabilization [10]. In this study, mean shoreline change rates varied significantly between Reserves, with the lower fetch (and likely wave energy) Currituck Banks exhibiting soundward movement of the marsh shoreline (Fig 2a), and the higher fetch Kitty Hawk Woods exhibiting landward movement of the marsh shoreline. These observations were consistent with the mean rates determined from the past two decades of shoreline imagery. Despite the lack of a significant difference in mean shoreline change rates between *Phragmites* Density, the finding of a greater rate of soundward expansion of the marsh shoreline in areas of High *Phragmites* Density in Currituck Banks relative to areas of Low *Phragmites* Density is consistent with studies in the Chesapeake Bay and the Netherlands [41, 42]. The Chesapeake Bay study identified a two-fold greater rate of sediment deposition associated with *Phragmites* marshes relative to native *Spartina* marshes [42]. In the Netherlands, reduced shoreline retreat rates and enhanced wave attenuation were observed in association with *Phragmites* stands [41]. The finding in the present study suggests this trend of greater shoreline stabilization with increasing *Phragmites* density may be dependent on the environmental setting (e.g., energy regime) of the marshes examined. We observed a greater (although not significant) mean landward shoreline retreat rate in areas of High *Phragmites* Density in Kitty Hawk Woods relative to areas of Low and Medium *Phragmites* Density. The Medium *Phragmites* Density sites had the lowest mean landward shoreline retreat rates relative to the other treatments, however, it is unclear whether this was anomalous or if the mix of *Phragmites* and native grasses provides a greater level of shoreline stabilization. Given the lack of a significant difference in mean shoreline change rates between *Phragmites* Density treatments within both Reserves, it appears that increasing *Phragmites* Density does not impact the ability of marshes we examined to provide shoreline stabilization services.

The capacity of marshes to store atmospheric carbon through primary production and vertical marsh accretion has generated considerable restoration and conservation interest [9, 10]. Mean normalized total below-ground carbon inventories did not vary significantly between Reserves or *Phragmites* Density treatments (Fig 3) despite a significant difference in above-ground biomass between Reserves (Fig 6). The higher energy environment of Kitty Hawk Woods likely yields greater suspension of marsh detritus (i.e., material derived from above-ground biomass) from the marsh platform and flux to the estuary than in the lower energy environment of Currituck Banks, and this process may explain the similar mean below-ground carbon inventories between the Reserves (sensu [43]). Within both Reserves, below-ground carbon inventories did not vary significantly by *Phragmites* Density due to the high degree of variability in below-ground carbon inventory within treatments. Although not significant, the greatest below-ground carbon inventories at Kitty Hawk Woods Reserve were observed at marshes with Low *Phragmites* densities, while High and Medium *Phragmites* marshes contained one-half and one-third, respectively, the below-ground carbon inventories of Low *Phragmites* marshes. The higher below-ground carbon inventories in the Low *Phragmites* Density site within Kitty Hawk Woods is consistent with the greatest observed above-ground biomass being present at this site. Within Currituck Banks, below-ground carbon inventories and above-ground biomass were approximately equivalent between *Phragmites* Density treatments. The lack of a significant difference in below-ground carbon inventories between Reserves or *Phragmites* Density treatments observed in this study appears to indicate that: (1) the below-ground biomass storage potential of marshes in both Reserves is similar, and (2) increasing *Phragmites* Density within each Reserve does not consistently impact the ability of marshes we examined to provide carbon storage services.

Plant diversity and community structure is a key factor underpinning the ability of marshes to deliver ecosystem services as it is often positively correlated with primary production, nutrient cycling, and resiliency to disturbance [11, 12]. In the present study, NMDS and ANOSIM results suggest that plant community composition varied significantly between Kitty Hawk Woods and Currituck Banks, but not by *Phragmites* Density within a Reserve. Despite the observed differences in plant composition between Reserves, plant species richness, evenness, and diversity (Simpson’s and Shannon’s) did not vary significantly between Reserves or by *Phragmites* Density. These findings directly contrast many previous studies that have identified a decrease in plant species richness, evenness and diversity in marshes invaded by *Phragmites* [18, 44].

One potential reason for the general neutral impact of *Phragmites* that we observed in the present study, may be the considerably lower above-ground biomass within the High *Phragmites* Density treatments relative to previous studies that examined *Phragmites* impacts (i.e., Fig 6b and 6c: ~250–500 g m^-2^ in the present study vs. 1,000–4,000 g m^-2^; [40] and references therein). Despite the relatively low above-ground biomass of *Phragmites* observed in the present study, the two Reserves provided a range of above-ground biomass over which to assess *Phragmites* impact. Mean above-ground biomass of *Phragmites* and *Spartina* was ~3.5x higher within Kitty Hawk Woods Reserve relative to Currituck Banks (Fig 6a: 963.78 ± 139.72 SE g dry plant material m^-2^ versus 271.46 ± 69.59), potentially as a result of more favorable growing conditions (e.g., nutrient availability) within Kitty Hawk Woods; however, this hypothesis requires testing. Increasing *Phragmites* Density was associated with a decrease in above-ground biomass within Kitty Hawk Woods, but not within Currituck Banks. Within Kitty Hawk Woods, the reduced above-ground biomass associated with High *Phragmites* Density was likely driven by both lower mean stem density (40.92 ± 8.50 stems m^-2^ versus 33.85 ± 8.13) and lower mean stem height (2.08 ± 0.30 m versus 1.98 ± 0.17) relative to Low *Phragmites* Density. This observation is consistent with an ~ 2x greater stem density within native *Spartina* marshes relative to invaded *Phragmites* marshes within Chesapeake Bay [45]. Within Currituck Banks, High *Phragmites* Density was associated with increased mean stem density (12.17 ± 3.97 stems m^-2^ versus 22.33 ± 6.58), but decreased mean stem height (1.52 ± 0.23 m versus 0.96 ± 0.28) relative to Low *Phragmites* Density. As a result, above-ground biomass was approximately equivalent between High and Low *Phragmites* Density treatments.

An important distinction between the marshes observed in this study and those observed in prior studies is their protected status as National Estuarine Research Reserves. For instance, previous studies that identified a decrease in the aforementioned plant community parameters have largely examined marshes impacted by shoreline development [44] or other forms of human disturbance (e.g., abandoned rice fields [18]). Disturbances can facilitate the spread of invasive species and can unpredictably affect native communities [46], therefore the diminished influence of human disturbance in these protected areas may explain the similarity in plant community parameters across *Phragmites* Density treatments. The reduced impact of human disturbance due to the protected status of the marshes examined in this study could explain the overall neutral effect of increasing *Phragmites* Density on the observed ecosystem services. Both Kitty Hawk Woods and Currituck Banks have been designated as protected, undeveloped wildlife preserves since the mid-to-late 1980s [47]. As a result, these marshes have not experienced shoreline development or habitat alteration that has previously been associated with aggressive invasion by *Phragmites* [15, 40, 44]. Furthermore, maintenance of undisturbed habitat through minimization of human impacts has previously been identified as a strong defense against invasive species [48].

Given the variable impacts of invasive species on ecosystem service provision [3, 4, 5] coupled with the significant costs associated with their management [2], understanding the density-dependent role of invasive species in the provision of essential ecosystem services can provide valuable information to support effective management. The generally neutral effect of increasing *Phragmites* Density observed in this study on ecosystem services suggests that costly and labor-intensive eradication efforts may not be justified if preservation of the ecosystem services we examined is an important management goal. Our study highlights the need for managers to critically evaluate what particular ecosystem service (or suite of services) should be prioritized when making decisions regarding invasive species management. Furthermore, given the discordance between some results of the present study and prior studies (e.g., impacts of increasing *Phragmites* Density on plant diversity), it is likely that the impact of increasing *Phragmites* Density on ecosystem services varies as a function of environmental setting. Thus, on-site monitoring of ecosystem services of management interest, especially along a gradient of invasive species density, is required to effectively integrate these considerations into site-specific management plans. The present study provides a framework for utilizing ecosystem service provision as the basis for developing a density-dependent threshold for *Phragmites* management, and this conceptual framework has broad implications for global management of invasive species using density-and ecosystem services-based approaches.

## Supporting information

S1 Fig Relationships between percent organic matter (determined via loss-on-ignition) and percent organic carbon (determined via CHN analysis) used to derive below-ground carbon inventory estimates. Reserve- and *Phragmites* Density-specific LOI versus organic carbon content relationships were developed. Kitty Hawk Woods: (A) High *Phragmites* Density, (B) Medium *Phragmites* Density, (C) Low *Phragmites* Density; Currituck Banks: (D) High *Phragmites* Density, and (E) Low *Phragmites* Density. *R*^*2*^ is provided as an estimate of model fit.
(TIFF)

S2 Fig Relationships between shoot height (m) and dry mass (g) used to derive above-ground biomass estimates. Plant Species- (*Phragmites* vs. *Spartina*) and *Phragmites* Density-specific shoot height versus dry mass relationships were developed: *Phragmites* in (A) Medium *Phragmites* Density, (B) High *Phragmites* Density; *Spartina* in (C) Medium *Phragmites* Density, and (D) Low *Phragmites* Density. Note that the Medium *Phragmites* Density treatment was present only in Kitty Hawk Woods Reserve. Standard error of the regression (S) is provided as an estimate of model fit.
(TIFF)

S1 Text Plant community structure: NDMS/ANOSIM analysis methods and interpretation. This document provides a detailed description of the statistical software and methods used for analyses of plant community structure data, including details about parameters, hypotheses tested, and interpretation of the non-metric multidimensional scaling and analysis of similarity analyses.
(DOCX)
