# Supplementary figures and images for: Density-dependent role of an invasive marsh grass, Phragmites australis, on ecosystem service provision
Source: PLoS One. 2017 Feb 24;12(2):e0173007. doi: 10.1371/journal.pone.0173007 (PMC5325552; doi:10.1371/journal.pone.0173007)

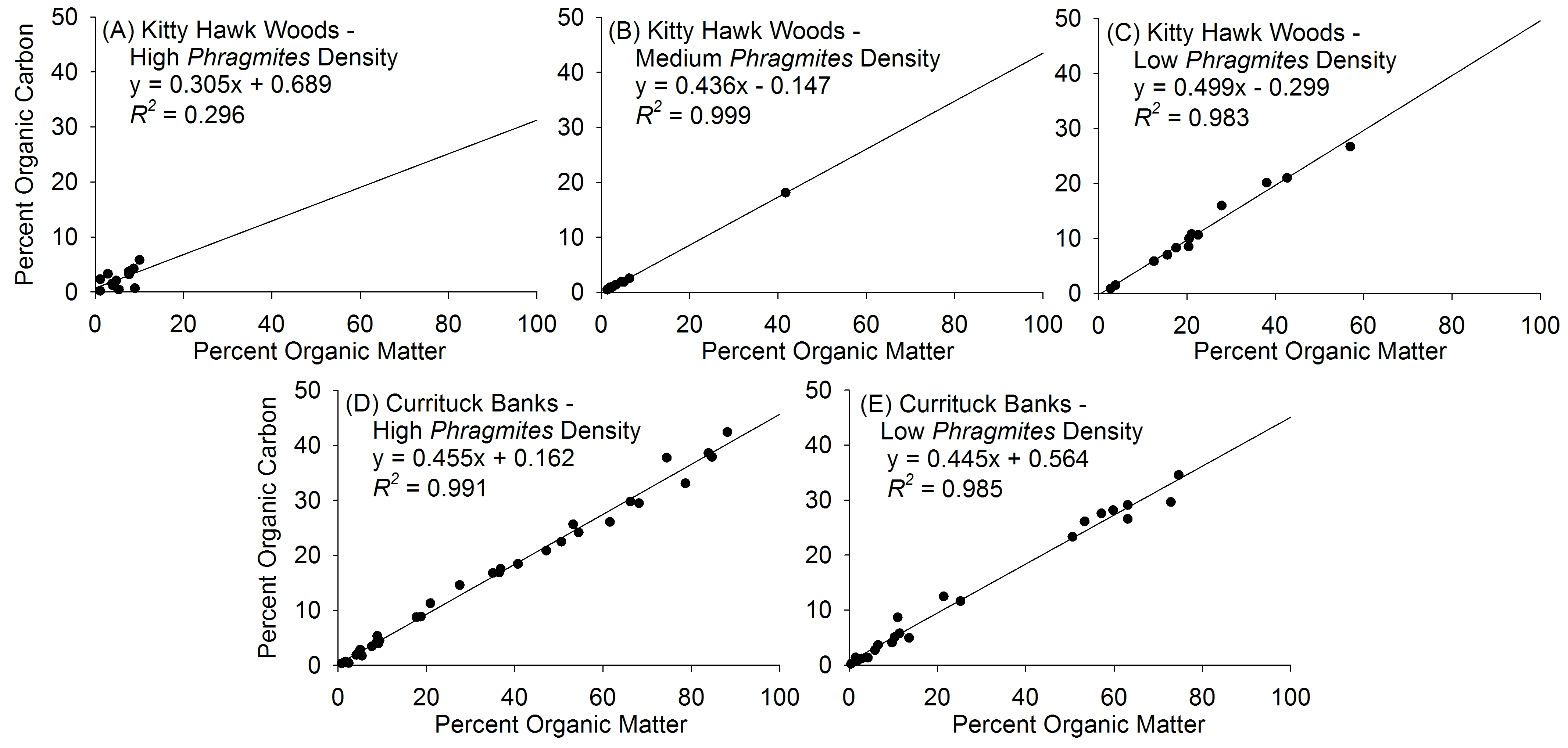

Supplement: S1 Fig — Reserve- and Phragmites Density-specific LOI versus organic carbon content relationships were developed. Kitty Hawk Woods: (A) High Phragmites Density, (B) Medium Phragmites Density, (C) Low Phragmites Density; Currituck Banks: (D) High Phragmites Density, and (E) Low Phragmites Density. R2 is provided as an estimate of model fit. (TIFF) [file pone.0173007.s001.tiff]

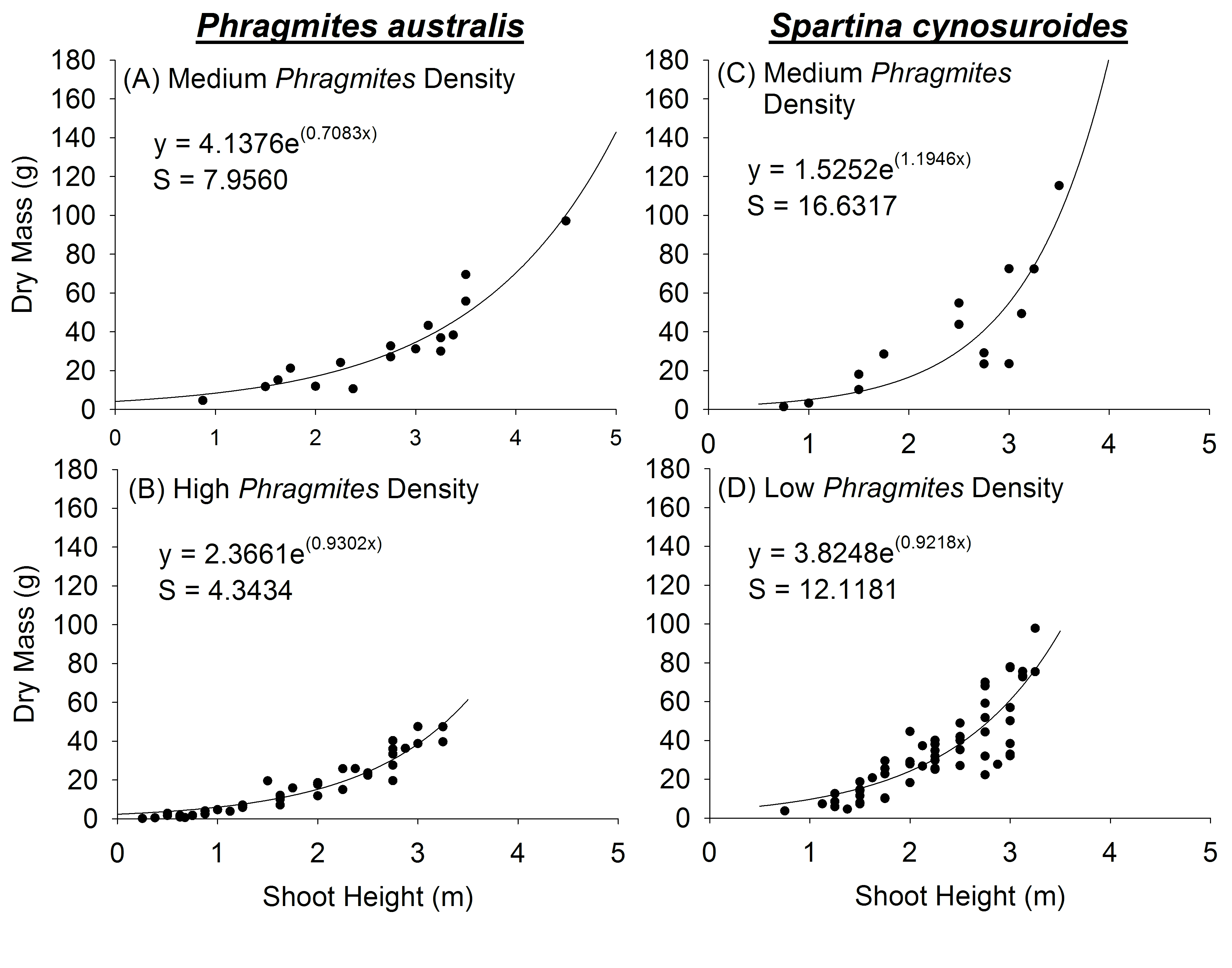

Supplement: S2 Fig — Plant Species- (Phragmites vs. Spartina) and Phragmites Density-specific shoot height versus dry mass relationships were developed: Phragmites in (A) Medium Phragmites Density, (B) High Phragmites Density; Spartina in (C) Medium Phragmites Density, and (D) Low Phragmites Density. Note that the Medium Phragmites Density treatment was present only in Kitty Hawk Woods Reserve. Standard error of the regression (S) is provided as an estimate of model fit. (TIFF) [file pone.0173007.s002.tiff]
