# Supplementary material for: Density-dependent role of an invasive marsh grass, Phragmites australis, on ecosystem service provision
Source: PLoS One. 2017 Feb 24;12(2):e0173007. doi: 10.1371/journal.pone.0173007 (PMC5325552; doi:10.1371/journal.pone.0173007)
Supplement: S1 Text — This document provides a detailed description of the statistical software and methods used for analyses of plant community structure data, including details about parameters, hypotheses tested, and interpretation of the non-metric multidimensional scaling and analysis of similarity analyses. (DOCX) [file pone.0173007.s003.docx]

**Supporting Information.**

**S1 Text.**

Plant community structure: NDMS/ANOSIM analysis methods and interpretation

A between-sample Bray-Curtis similarity matrix was created using the percent cover data for each plant species (with the exception of *Phragmites* and *Spartina*, as described in ‘Methods’) within each Reserve and *Phragmites* Density treatment. We utilized non-metric multidimensional scaling (NMDS) with 999 permutations and 100 random starts within PRIMER v7 to depict similarities and differences in the community structure within each Reserve and *Phragmites* Density treatment type (Clarke and Gorley 2015). Analysis of similarity (ANOSIM) was performed on the Bray-Curtis similarity matrix (999 permutations) to determine if community composition significantly varied based on Reserve or *Phragmites* density type.

As NMDS uses rank orders of similarities between assemblages, there are no restrictive assumptions of normality, and thus is a robust ordination technique for community analysis (Clarke et al. 2014). Our NMDS plot is presented with a two-dimensional ordination stress value, which indicates the degree of mismatch between the predicted values from the regression of the similarity matrix and the distances between samples as displayed by the two-dimensional nMDS plot. Stress values less than 0.20 generally indicate an interpretable ordination (Clarke 1993).

ANOSIM was used to test the null hypotheses of no differences between plant community assemblages by Reserve of *Phragmites* Density treatment. The significance levels, including the p-value and R (an indicator of separation between groups; higher values indicate greater differences in plant community assemblages), were determined by random permutations of each Bray-Curtis matrix.

References:

Clarke KR. Non-parametric multivariate analyses of changes in community structure.

Austral Ecology. 1993;18: 117–143.

Clarke KR, Gorley RN. PRIMER v7: User Manual/Tutorial. 7^th^ ed. Plymouth: PRIMER-

E; 2015.

Clarke KR, Gorley RN, Somerfield PJ, Warwick RM. Change in marine communities: an

approach to statistical analysis and interpretation. 3^rd^ ed. Plymouth: PRIMER-E; 2014.
